# Time course and side-by-side analysis of mesodermal, pre-myogenic, myogenic and differentiated cell markers in the chicken model for skeletal muscle formation

**DOI:** 10.1111/joa.12353

**Published:** 2015-08-17

**Authors:** Federica Berti, Júlia Meireles Nogueira, Svenja Wöhrle, Débora Rodrigues Sobreira, Katarzyna Hawrot, Susanne Dietrich

**Affiliations:** 1 Institute for Biomedical and Biomolecular Science (IBBS), School of Pharmacy and Biomedical Sciences, University of PortsmouthPortsmouth, UK; 2 Instituto de Ciências Biológicas, Departamento de Morfologia, Universidade Federal de Minas Gerais (UFMG)Belo Horizonte, Minas Gerais, Brazil; 3 Department of Human Genetics, University of ChicagoChicago, IL, USA

**Keywords:** *Cdh4*, chicken embryo, dermomyotome, *Desmin*, *Eya1*, *Follistatin*, *Mef2* genes, *Mrf4*, *Myf5*, *Myh15*, *Myh7*, *MyoD*, *MyoG*, myotome, paraxial mesoderm, *Paraxis*, *Pax3*, *Pax7*, *Pitx3*, sarcomeric *Myosin*, *Six1*, skeletal muscle, somite, *Tbx6*, *Tnni1*

## Abstract

The chicken is a well-established model for amniote (including human) skeletal muscle formation because the developmental anatomy of chicken skeletal muscle matches that of mammals. The accessibility of the chicken in the egg as well as the sequencing of its genome and novel molecular techniques have raised the profile of this model. Over the years, a number of regulatory and marker genes have been identified that are suited to monitor the progress of skeletal myogenesis both in wildtype and in experimental embryos. However, in the various studies, differing markers at different stages of development have been used. Moreover, contradictory results on the hierarchy of regulatory factors are now emerging, and clearly, factors need to be able to cooperate. Thus, a reference paper describing in detail and side-by-side the time course of marker gene expression during avian myogenesis is needed. We comparatively analysed onset and expression patterns of the key markers for the chicken immature paraxial mesoderm, for muscle-competent cells, for cells committed to myogenesis and for cells entering terminal differentiation. We performed this analysis from stages when the first paraxial mesoderm is being laid down to the stage when mesoderm formation comes to a conclusion. Our data show that, although the sequence of marker gene expression is the same at the various stages of development, the timing of the expression onset is quite different. Moreover, marker gene expression in myogenic cells being deployed from the dorsomedial and ventrolateral lips of the dermomyotome is different from those being deployed from the rostrocaudal lips, suggesting different molecular programs. Furthermore, expression of Myosin Heavy Chain genes is overlapping but different along the length of a myotube. Finally, *Mef2c* is the most likely partner of Mrf proteins, and, in contrast to the mouse and more alike frog and zebrafish fish, chicken *Mrf4* is co-expressed with *MyoG* as cells enter terminal differentiation.

## Introduction

Vertebrates evolved from their chordate ancestors 550 million years ago (reviewed in Clack, 2002). These animals – as well as all extant non-vertebrate chordates – lived in an aqueous environment. Accordingly, their mode of locomotion was swimming via undulating movements of the body and tail, which created a thrust against the water. The anatomical basis of chordate movements are segmented blocks of skeletal muscle, or myotomes, on either side of the central skeletal element, the notochord. In vertebrates, eventually the vertebral column functionally replaced the notochord, and muscle pattern became more complex. However, in the embryo, notochord and myotomes are present. In vertebrates that develop via free-feeding larvae (all vertebrate taxa with the exception of amniotes), the myotomes become immediately functional and allow the larva to swim in a similar fashion as larvae of non-vertebrate chordates. During the transition to adulthood, myotomal muscles regroup. This is particularly advanced in tetrapods, where myotomal muscles are rearranged to provide the back and abdominal muscles, essential to lift the body off the ground, and in humans, to stand upright. However, the segmental organisation of muscle is still evident for the intercostal muscles.

Myotomes and notochord are derived from the embryonic middle germ layer, the mesoderm (reviewed in Gilbert, 2000). As the notochord demarcates the longitudinal axis of the developing embryo, the neighbouring myotomes are also referred to as *par*-axial mesoderm (reviewed in Bryson-Richardson & Currie, 2008; Buckingham & Vincent, 2009; Relaix & Zammit, 2012). Significantly, during vertebrate evolution, additional paraxial mesodermal cell types evolved. Already during the evolution of chordates, animals acquired the ability to generate muscle stem cells for later phases of muscle growth and repair (Holland et al. 1999; Somorjai et al. 2012). In vertebrates, these cells are transiently stored in a compartment dorsolateral to the myotome. The cells initially also have the ability to contribute to the dorsal dermis, and this compartment is therefore referred to as dermomyotome. During the evolution of the vertebrate lineage, cells in the paraxial mesoderm also acquired the ability to form the cartilages and bones of the vertebral column and ribs. These cells are also allocated to a separate compartment, the ventrally located sclerotome (which includes precursors for muscle connective tissue and tendons collectively referred to as syndetome). We thus refer to the vertebrate segmented paraxial mesoderm as somites, which subdivide into sclerotome, dermomyotome and a myotome placed in between.

Differentiated, functional muscle consists of postmitotic cells. In the amniotes, adult muscle mass increases via hypertrophy, the generation of more contractile proteins. During embryonic, fetal and perinatal phases of development, muscle grows via hyperplasia, the addition of cells from the mitotically active muscle precursor/muscle stem cell pool. This occurs in waves (reviewed in Buckingham & Vincent, 2009; Relaix & Zammit, 2012 for amniotes; for anamniotes see Bryson-Richardson & Currie, 2008). In amniotes, first cells from the medial wall of the newly formed somites spread laterally between the emerging sclerotome and dermomyotome. They form the primary myotome that provides a scaffold for the cells arriving next (Kahane et al. 1998b). Then, cells from the dorsomedial lip of the dermomyotome detach and contribute to the myotome, thereby driving its dorsomedial outgrowth (Denetclaw et al. 1997). With a slight delay, cells from the ventrolateral dermomyotomal lip engage in the same process, driving ventrolateral outgrowth (Kahane et al. 1998a, 2007; Denetclaw & Ordahl, 2000; Pu et al. 2013); the exception is found at limb levels where cells from the ventrolateral dermomyotomal lip emigrate to provide the limb musculature. Cells from the rostral and caudal lips also detach and intercalate into the existing scaffold, thereby driving the extension of the myotomal centre (Kahane et al. 1998a). Together, this second wave of cell influx leads to an elongated, secondary myotome. Eventually, the centre of the dermomyotome disperses, and cells enter the myotome directly (Kahane et al. 2001; Ben-Yair & Kalcheim, 2005; Gros et al. 2005; Relaix et al. 2005; Ahmed et al. 2006). These cells, while losing the ability to become dermis, retain their stem cell features, being able to self-renew and to produce differentiating daughters. Thus, these cells are specialised muscle stem cells which provide the – now tertiary – myotome with an innate ability to enlarge (Hutcheson et al. 2009). In the adult, the muscle stem cells adopt a quiescent state, only to become activated when muscle is in need of repair (reviewed in Relaix & Zammit, 2012).

A number of regulatory genes have been associated with the process of myogenic cell deployment and differentiation. When the paraxial mesoderm is being laid down during gastrulation, it expresses the T-box transcription factor Tbx6 and the axial mesoderm expresses its paralog T (Brachyury); *Tbx6* has been shown to be essential for paraxial mesoderm development and the suppression of premature differentiation (Chapman & Papaioannou, 1998; Chapman et al. 2003; Windner et al. 2012). As the paraxial mesoderm segments, genes primarily associated with Notch-Delta signalling but also other signalling systems are being expressed in a cyclic fashion and control segment periodicity. Controlled by a caudal-high to rostral-low Fgf gradient, cyclic gene expression stops, and gene expression is stabilised in the prospective rostral or caudal compartment of the developing somite (reviewed in Hubaud & Pourquie, 2014).

In amniotes, simultaneous with the arrest of cyclic gene expression, signals from the surface ectoderm trigger the expression of the bHLH transcription factor Paraxis, and the two paralogous paired box transcription factors Pax3 and Pax7, which facilitate epithelial somite formation and segregation of somites from the as yet not segmented paraxial mesoderm (segmental plate, pre-somitic mesoderm; Burgess et al. 1996; Šošic et al. 1997; Dietrich et al. 1997; Mansouri & Gruss, 1998; Schubert et al. 2001; Linker et al. 2005). Importantly, *Paraxis* and the two *Pax* genes remain expressed in the dermomyotome while being downregulated (*Paraxis*) or shut off (*Pax3*, *Pax7*) in the sclerotome; specifically *Pax7* remains expressed in muscle stem cells throughout life. Mutations for these genes cause muscle or muscle regeneration defects *in vitro* and *in vivo*, and it has been shown for Pax3/7 that these factors can, at least in certain genetic contexts, directly bind and upregulate expression of *MyoD*, a key gene associated with myogenic commitment (see below; Tajbakhsh et al. 1997; Tremblay et al. 1998; Wilson-Rawls et al. 1999; Seale et al. 2000; Relaix et al. 2004, 2006; Collins et al. 2009; Lepper et al. 2009; Hutcheson et al. 2009; von Maltzahn et al. 2013; reviewed in Relaix & Zammit, 2012). Therefore, *Paraxis* and *Pax3/Pax7* are referred to as pre-myogenic genes.

Parallel to the *Pax* genes, *Six* genes, *Dach* genes (negative Six regulators) and *Eya* genes (positive Six regulators) have been implicated in the initiation of myogenesis (Heanue et al. 1999; reviewed in Aziz et al. 2010). Single and double knockout experiments have shown that paralogous *Six* and *Eya* genes have overlapping roles. Important for this study, mammalian *Six* genes both of the *sine oculis* (*Six1, 2*) and the *Six4* (*Six4, 5*) but not the *optix* (*Six3,6*) subfamily are expressed in somites. Moreover, they also can bind to the promoters of *MyoD* and the related *MyoG* gene and upregulate their expression (Spitz et al. 1998; Relaix et al. 2013). Thus, *Six* and *Eya* genes are also referred to as pre-myogenic genes. In the ventrolateral lips of the mouse dermomyotome, *Pax3* expression is lost when *Six* and *Eya* genes are mutated (Grifone et al. 2005, 2007). Thus, in this case, *Six* and *Eya* genes act upstream of the *Pax* genes. On the other hand, *Six* and *Eya* genes regulate the expression of genes required for the establishment of the fast-twich, glycolytic muscle fibre type, hence displaying a prolonged role downstream in myogenesis (Grifone et al. 2004).

The MyoD family of basic helix-loop-helix transcription factors is firmly associated with myogenic commitment, and is referred to as Mrf (muscle regulatory factors; reviewed in Aziz et al. 2010; Fong & Tapscott, 2013). *In vitro*, any of the four paralogous genes – *Myf5*, *MyoD*, *MyoG* (*Myogenin*, *Mng*) and *Mrf4* (*Myf6, Herculin*) – can drive myogenic as well as non-myogenic cells into myogenic differentiation (Braun et al. 1989b; Edmondson & Olson, 1989; Weintraub et al. 1989; Miner & Wold, 1990). Moreover, Mrf bind to promoters of numerous muscle differentiation genes (Cao et al. 2010). However, *Myf5* and *MyoD* are expressed early and in cells that are still mitosis-competent; in the mouse, the double knock-out prevents the formation of myoblasts. On the other hand, *Myogenin* is expressed when cells withdraw from the cell cycle and enter terminal differentiation, and in knock-out mice, myoblasts form but fail to become differentiating myocytes (Rawls et al. 1995; Wang & Jaenisch, 1997; Bergstrom & Tapscott, 2001). Yet *MyoD* and *MyoG* function is linked in a feed-forward mechanism, with *MyoD* upregulating its own expression and that of *MyoG*, and both cooperate to activate muscle structural genes (Penn et al. 2004; Cao et al. 2006). *Mrf4* is arguably the most dubious *Mrf* as, phylogenetically, it is most closely related to *MyoG*. However, in the mouse, expression commences early and is required for hypaxial myogenesis from the ventrolateral dermomyotomal lips (Atchley & Fitch, 1997; Summerbell et al. 2002; Kassar-Duchossoy et al. 2004; Zheng et al. 2009). However, its main expression phase is during fetal myogenesis.

During the initiation of myogenesis, Mrf bind to target promoters at sites closely linked to the binding sites of MADS box transcription factors of the Myocyte Enhancer Factor 2 family (Mef2a,b,c,d; reviewed in Naya & Olson, 1999). These factors, while poorly promoting myogenesis alone, enhance the myogenic capacity of the Mrf (Molkentin et al. 1995). Moreover, Mrf and Mef2 factors physically interact (Black et al. 1998), and they enhance each other's expression in positive feedback loops (Braun et al. 1989a; Edmondson et al. 1992). However, *Mef* genes also have a role in the differentiation of cardiomyocytes (and other tissues), whereas *Mrf* gene function is restricted to skeletal muscle (reviewed in Wu et al. 2011).

Eventually, the activation of the myogenic cascade cumulates in the activation of muscle structural genes that are crucial for the functional properties of the cells (reviewed in Alberts et al. 1983). Among these are genes that control the elongation of myocytes into myotubes, and the establishment of protein complexes (sarcomeres) that control cell contraction. Moreover, myotubes will align, and they will recruit cells to fuse into terminally differentiated syncytial myofibres (reviewed in Abmayr & Pavlath, 2012; Hindi et al. 2013). Thus, expression of cell adhesion molecules and of sarcomeric proteins such as muscle Actin, Troponin, Tropomyosin, muscle Myosin and the Z-line protein Desmin are indicators of the terminal differentiation process.

The above outline suggests that amniote myogenesis is governed by a stereotypical, sequential action of regulatory genes. However, the various waves of myogenesis suggest that the gene regulatory cascades are not equivalent during these phases. Likewise, the dermomyotomal lips and the dermomyotomal centre are not equivalent sources of myogenic cells. Furthermore, studies in P19 embryonic carcinoma cells indicated that MyoD can act upstream of the premyogenic genes (Gianakopoulos et al. 2011). Epigenetic studies showed that MyoD, Mef2 and Six proteins have to interact with each other and with histone modifying enzymes that control the opening of chromatin in order to activate target genes (reviewed in Aziz et al. 2010; Fong & Tapscott, 2013). This suggests that regulatory networks are complex, and cellular decisions depend on which factors are available at a given time.

Owing to its large size, extra-uterine development, ease of manipulation and low costs, the chicken has always been the model of choice for embryological studies of amniote muscle development. Moreover, understanding chicken muscle development in its own right is important for the poultry industry. Nonetheless, its long generation time and large size of the adults has rendered the chicken unsuited for genetic studies. However, following the sequencing of the chicken genome and the establishment of a variety of novel methods for transient genetic and genomic manipulation, the popularity of this model is picking up momentum (reviewed in Burt, 2007; Cogburn et al. 2007). A growing number of gene expression patterns are being deposited in the GEISHA (Gallus Expression *In Situ* Hybridization Analysis) database (http://geisha.arizona.edu). Astonishingly, myogenic gene expression has not been systematically analysed and, for example, it is not known where premyogenic and myogenic gene expression may overlap, and which of the four *Mef2* genes might be the most prominent partner of *Mrfs* in the chicken myotome. Moreover, *Myf5*, *MyoD* and sarcomeric Myosin expression have all been used as markers for myogenic differentiation, making the comparison of experimental data difficult.

To address this problem, we have comparatively analysed onset and expression patterns of the key markers for the chicken immature paraxial mesoderm, for muscle-competent cells, for cells committed to myogenesis and for cells entering terminal differentiation, focusing on the stages when somites and, subsequently, the primary and secondary myotome form. Our data reveal a set sequence of gene expression, yet the timing of expression onset was quite different at different stages of development. Moreover, marker gene expression in myogenic cells being deployed from the dorsomedial and ventrolateral lips of the dermomyotome was different from cells being deployed from the rostrocaudal lips, suggesting different molecular programs. Furthermore, expression of Myosin Heavy Chain genes overlapped but differed along the length of a myotube. Finally, our work revealed that *Mef2c* is the most likely partner of Mrfs, and, in contrast to the mouse and more akin to the frog and zebrafish fish models, chicken *Mrf4* did not show an expression phase prior to that of *MyoD*; instead, it was co-expressed with *MyoG* as cells entered terminal differentiation.

## Material and methods

### Culture and staging of embryos

Fertilised chicken eggs from a mixed flock (Winter Farm, Royston, and Henry Stewart Ltd, Norfolk, VA, USA) were incubated in a humidified atmosphere at 38.5 °C and staged according to (Hamburger & Hamilton, 1951). Embryos were harvested in 4% paraformaldehyde.

### *In situ* hybridisation

Whole mount *in situ* hybridisation and double *in situ* hybridisation was carried out as described by (Dietrich et al. 1997, 1998, 1999). Probes are detailed in Table S1.

### Immunohistochemistry

Whole mount antibody staining and antibody staining following an *in situ* hybridisation were carried out as described by Mootoosamy & Dietrich (2002), Alvares et al. (2003) and Lours & Dietrich (2005). For the anti-Desmin, -Myh7, -Myh15 and -Tnni1 antibody staining, a heat-induced epitope retrieval (HIER) was performed for 30 min at 95 °C, using 10 mm Tris pH9, 1 mm EDTA, 0.05% Tween-20 (anti-Desmin antibody) or 1.8 mm citric acid, 8.2 mm sodium citrate, 0.05% Tween at pH5 (anti-Myh7, -Myh15 and -Tnni1 antibodies). Details of the antibodies can be found in Supporting Information Table S2.

### Vibratome sectioning

Embryos subjected to whole mount stainings were embedded in 20% gelatine and cross-sectioned to 30–50 μm on a Pelco 1000 Vibratome as described in Dietrich et al. (1997, 1998, 1999).

### Photomicroscopy

Images in Fig. 7G–K are flattened z-stacks acquired on a Zeiss LSM710 confocal microscope. All other embryos and sections were photographed on a Zeiss Axioskop, using Nomarski optics. Images were acquired using the axiocam/axiovision system. All images were processed using adobe photoshop.

## Results

**Fig. 1:**
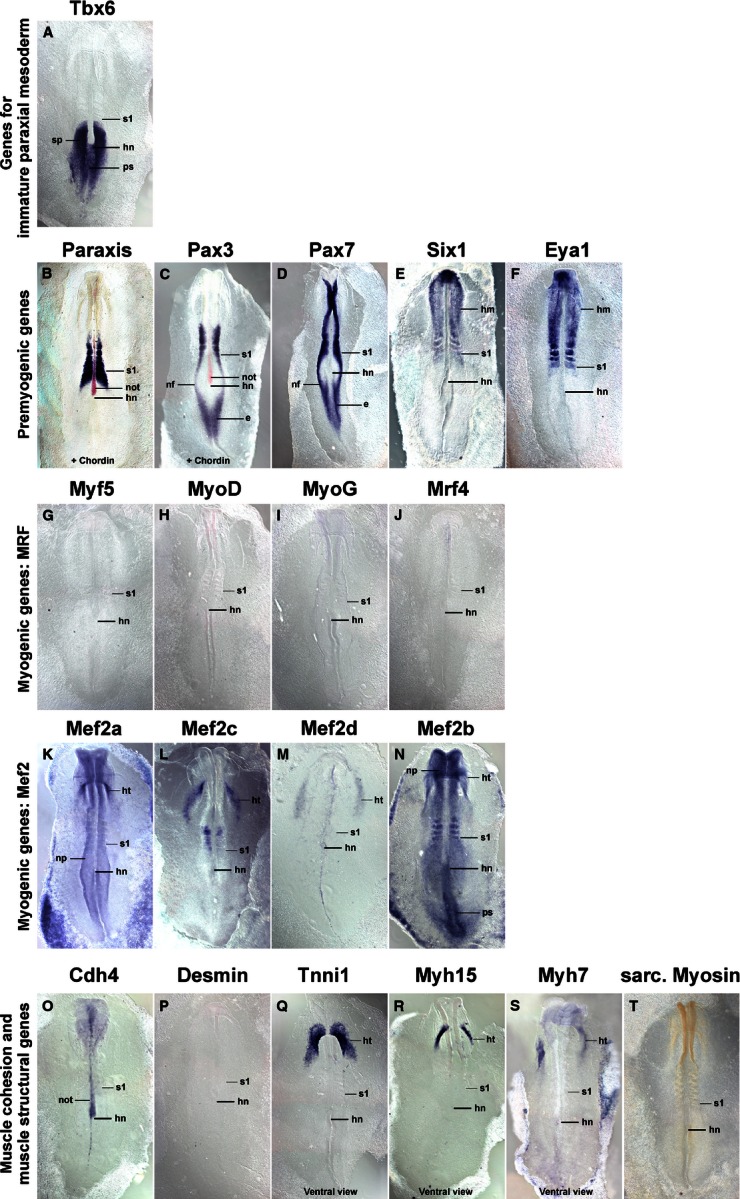
Marker gene expression at HH8. Dorsal views (Q–S: ventral views) of chicken embryos with three to five somites, rostral to the top. Markers are shown on top of each individual image. (B,C) The notochord is additionally stained for *Chordin* expression in red. Note the overlapping expression of *Tbx6* and the pre-myogenic genes in the rostral segmental plate and most recently formed somite (s1). *Mrf* genes are not yet expressed. *Mef2c* and *2d* display some somitic expression. However, the main expression of *Mef2* genes, and of *Tnni1*, *Myh15*, *Myh7* is in heart precursors (ht). e, epiblast; hm, head mesoderm; hn, Hensen's node; ht, cardiac precursors; nf, neural folds; not, notochord; np, neural plate; ps, primitive streak; s, somite; sp, segmental plate; the position of the youngest somite (s1) is indicated.

**Fig. 2:**
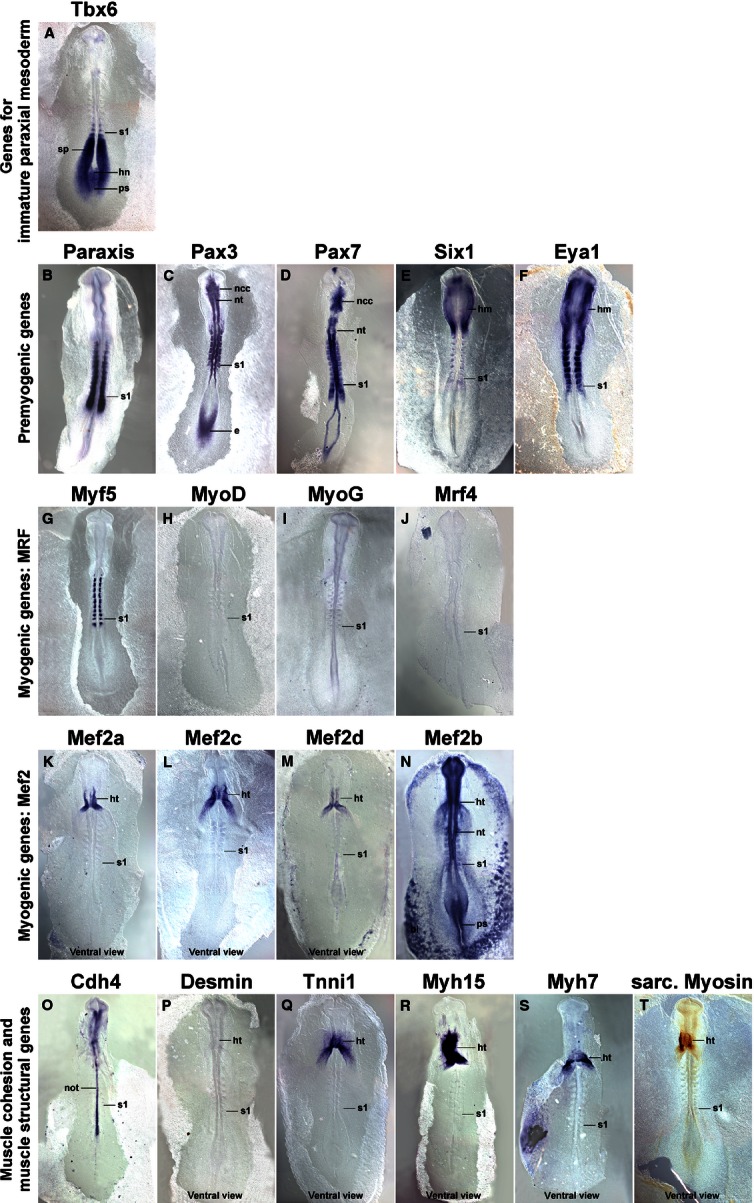
Marker gene expression at HH10. Dorsal views (P–T: ventral views), rostral to the top. Markers are indicated on top of each individual image as before. *Tbx6* and the pre-myogenic genes show overlapping expression in the rostral segmental plate and the most recently formed somite. The pre-myogenic genes label the condensing as well as fully formed epithelial somites. Of the *Mrf* genes, *Myf5* is expressed weakly in the condensing somite, and more robustly in the medial wall of the epithelial somites. Similar to HH8, *Mef2c* and *2d* display some weak somitic expression, but the main expression of the *Mef2* genes, of *Tnni1* and the Myosins remains in heart (ht). Abbreviations see Fig. 1 and: bi, blood islands; ncc, neural crest cells; nt, neural tube; the position of the youngest somite is indicated (s1).

**Fig. 3:**
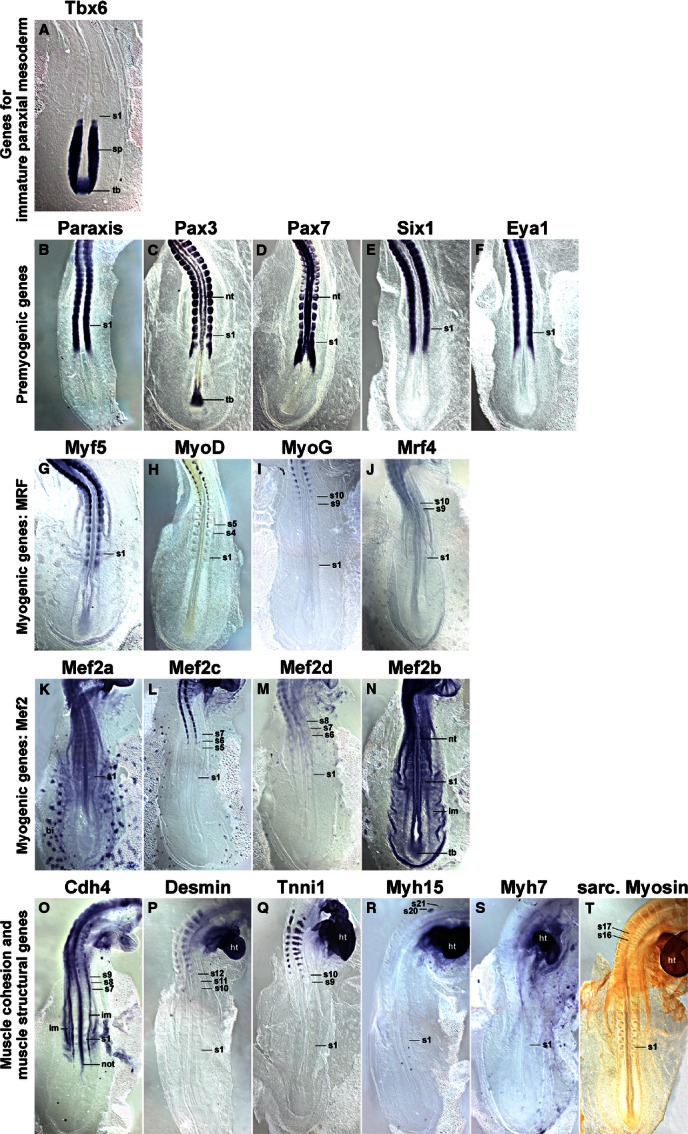
Marker gene expression at HH14. Dorsal views of the caudal region of HH14 chicken embryos, rostral to the top. Similar to earlier stages, *Tbx6* expression and the expression of pre-myogenic genes and of *Myf5* overlaps in the rostral segmental plate and youngest somite. More mature somites sequentially express *MyoD*, *Mef2c, Mef2d, Cdh4, MyoG, Mrf4, Tnni1, Desmin* and the Myosins (exception: *Myh7*; not yet expressed). Abbreviations see Figs 1,2 and: im, intermediate mesoderm; lm, lateral mesoderm; tb, tail bud; the position of the youngest somite is indicated (s1).

**Fig. 4:**
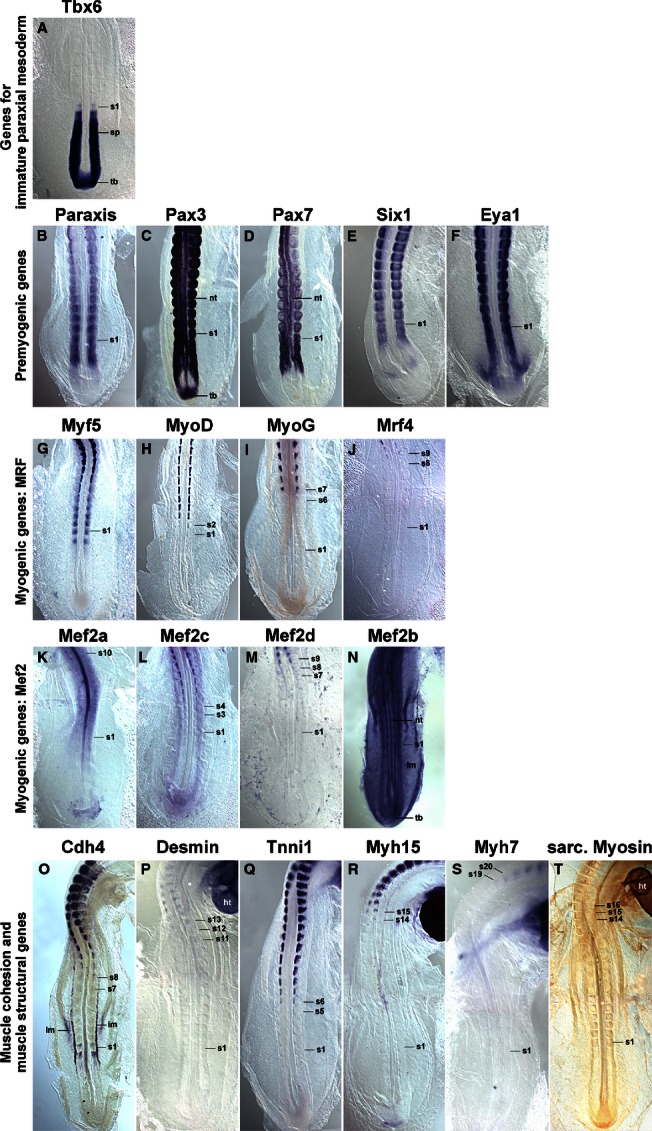
Marker gene expression at HH16. Dorsal views of the caudal region of HH16 chicken embryos, rostral to the top. Expression patterns are similar to those at HH14; however, expression of *MyoD*, *MyoG*, *Mrf4*, *Mef2c*, *Mef2d* and the muscle structural genes begins earlier. *Myh7* is now also expressed; elevated expression in the myotome of somite 10 is visible for *Mef2a*. Abbreviations and annotations as in Figs 1–3.

**Fig. 5:**
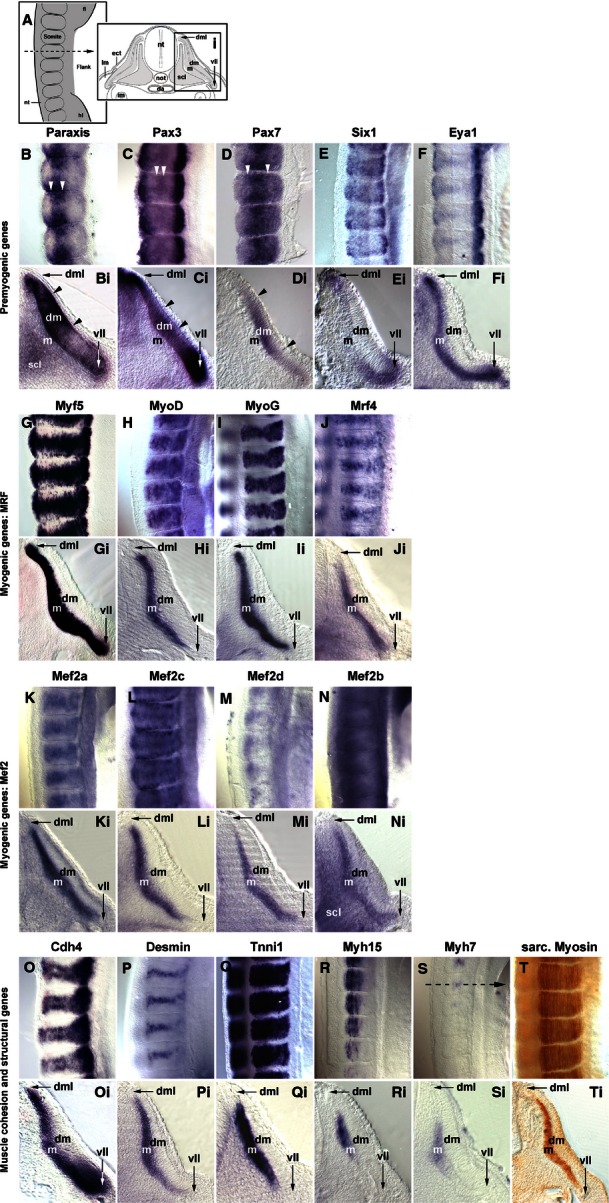
Marker gene expression in the flank of embryos at HH19-20. (A) Schematic representation of the images displayed in B–T (lateral view of flank somites on the right of the embryo, rostral to the top, lateral to the right) and Bi–Ti [cross section to flank somites, dorsal to the top, lateral to the right; section (Si) is from the forelimb- flank boundary as indicated in S]. Markers are indicated as before. *Paraxis*, *Pax3* and *Pax7* show distinct areas of elevated expression in the dermomyotome (B, Bi–D, Di; arrowheads). Their expression overlaps in dorsomedial and ventrolateral lips with that of *Six1*, *Eya1* and *Myf5*; in the ventrolateral lip, expression overlaps also with that of *Cdh4*. The *Mrf* genes, the *Mef2* genes and the genes encoding cell adhesion and muscle structural proteins show overlapping expression in the myotome, with the late commencing markers still being confined to the more medial territories. Abbreviations (see also Figs 1–3): da, dorsal aorta; dm, dermomyotome; dml, dorsomedial lip of dermomyotome; ect, surface ectoderm; fl, fore limb; hl, hind limb; m, myotome; scl, sclerotome; vll, ventrolateral lip of dermomyotome.

**Fig. 6:**
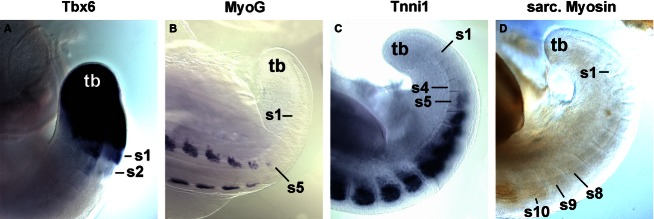
Expression of selected markers at the caudal end of HH19-20 embryos. Dorsolateral views of the caudal end of HH19-20 embryos; the position of the tail bud (tb) is indicated. Marker genes are indicated above the individual images as before. Similar to earlier stages, *Tbx6* expression still continues in the recently formed somites. However, the onset of *MyoG*, *Tnni1* and sarcomeric Myosin expression occurs significantly earlier, i.e. closer to the tail bud. Annotations as before.

**Fig. 7:**
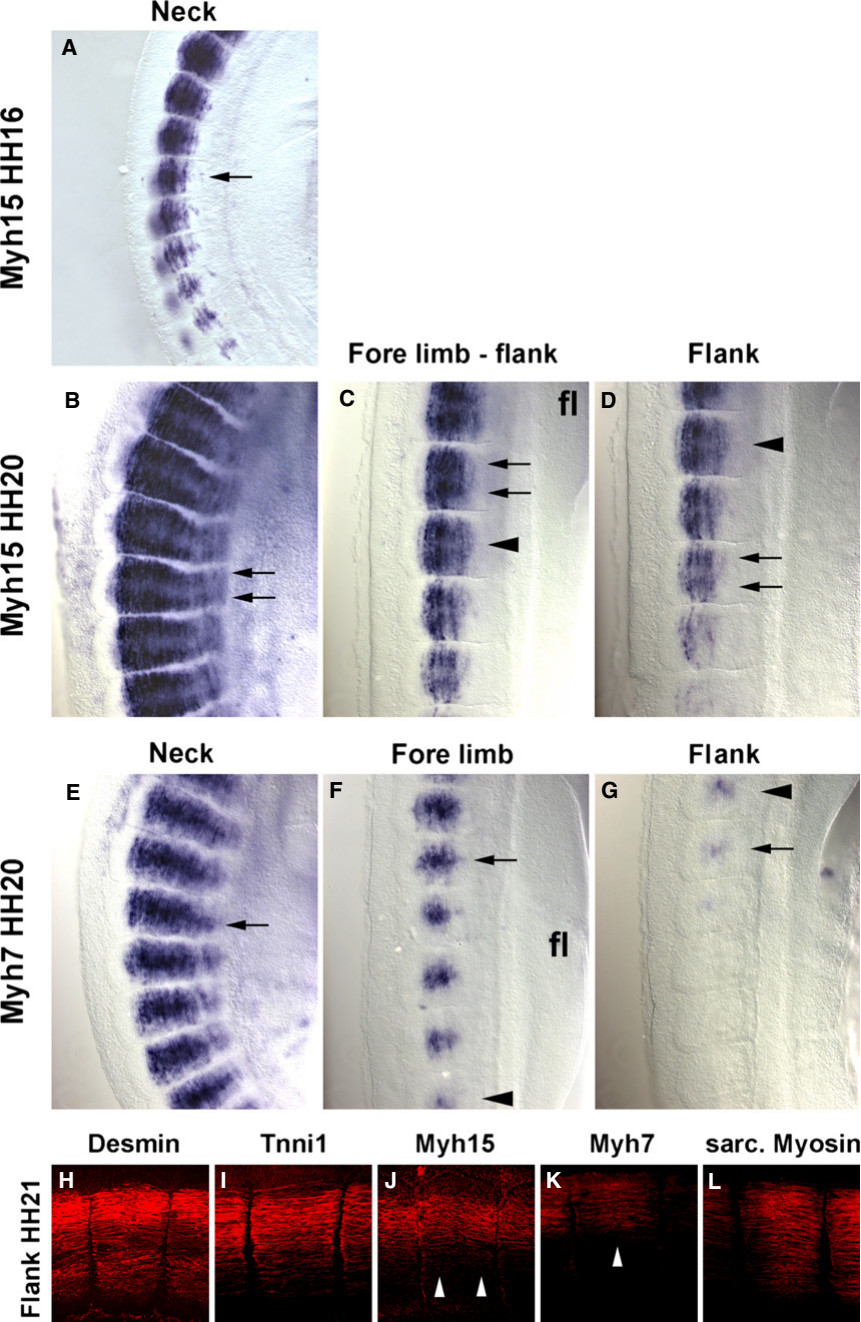
Comparison of *Myh15* and *Myh7* expression at HH16, 20 and 21. (A–D) *Myh15* mRNA expression in the somites of the HH16 neck (A) and the HH20 neck (B), fore limb-flank border (C) and the flank (D). (E–G) *Myh7* mRNA expression in the HH20 neck (E), at fore limb levels (F) and in the flank (G). Anterior is to the top in all, dorsal to the left. Arrowheads mark the same somites in (C,D) and (F,G), respectively. Note that at HH16, *Myh15* expression is strongest in the centre of the developing myotubes; at HH20, expression is strongest at the rostro-caudal ends (arrows), whereas *Myh7* labels the centres of the myotubes (arrows). (H–L) Flattened confocal z-stacks of HH21 flank somites, stained with antibodies detecting the proteins indicated on the top of the panel. Lateral views, dorsal to the top, anterior to the right. Note that Myh15 protein accumulated more strongly along the rostro-caudal edges of the myotome, whereas Myh7 protein is more concentrated in the centre (arrowheads). Annotations as before.

**Fig. 8:**
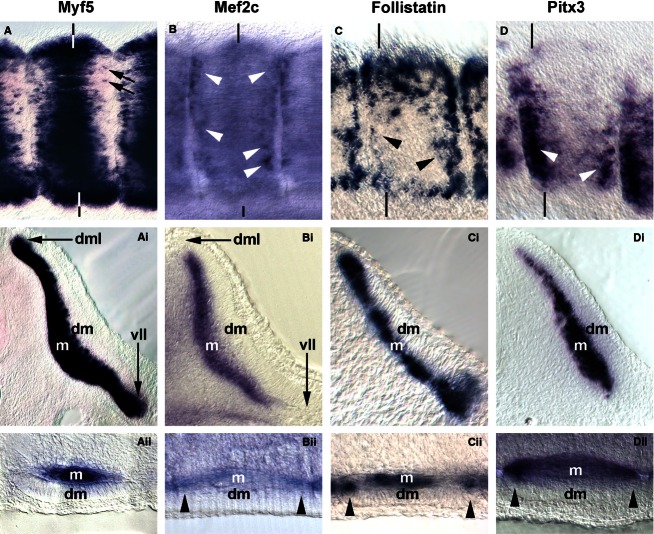
Comparison of markers labelling myogenic cells from the dorsomedial-ventrolateral and rostrocaudal lips of the dermomyotome. (A–D) Lateral views of flank somites on the right of the embryo, rostral to the right, dorsal to the top. (Ai–Di) Cross sections of these somites; (Ai, Bi) leading through the centre; (Ci,Di) sectioned along the caudal edge of the somite as indicated by the vertical lines. (Aii–Dii) Frontal sections, medial to the top, rostral to the right. Individual cells along the rostrocaudal sub-lip domain of the myotome express *Myf5* (A, arrows). In contrast, robust and widespread expression in this domain is found for *Mef2c*, *Follistatin* and *Pitx3* (B–D, Bii–Dii; arrowheads).

Since the myotome develops from somites, we focused our analysis on the period between stage HH4-5 when the primitive streak begins to lay down the prospective somitic mesoderm, HH7-8 when the first somites emerge, and HH19-20 when almost all of the 50 chicken somites have been generated (Hamburger & Hamilton, 1951). Gene expression was monitored by *in situ* hybridisation; to detect sarcomeric Myosins, the MF20 antibody was also used. As most markers were not expressed at HH4-5, embryos are not shown. The onset of gene expression is shown side-by-side for HH8 (Fig. 1), HH10 (Fig. 2), HH14 (Fig. 3) and HH16 (Fig. 4). Expression in mature flank somites of HH19-20 embryos is shown side-by-side in Fig. 5; additional, detailed marker comparisons at HH19-20 are displayed in Figs 6–8. The developmental age of somites was determined as in McGrew & Pourquie (1998), counting the condensing somite as somite 0, the first fully formed somite as somite 1, the next as somite 2, etc. Results are summarised in Table 1.

**Table 1:** Maturation age of the paraxial mesoderm expressing a gene at selected stages of development

Stage	HH4/5	HH8	HH10	HH14	HH16	HH19/20	Comments
Key feature	Fully extended primitive streak – streak beginning to retract, head process visible	4 somites	10 somites	22 somites	26–28 somites	37–43 somites	
Gene							
*Tbx6*; expression encompasses	*n* = 2; ps/ emerging mesoderm	*n* = 3; ps, sp, s0	*n* = 5; ps, sp, s0–s2/3	*n* = 5; tb, sp, s0	*n* = 5; tb, sp, s0–s1	*n* = 3; tb–s1/2	
Onset							
*Paraxis*	*n* = 3; condensing somite	*n* = 2; rostral sp, s0	*n* = 10; rostral sp, s0	*n* = 3; rostral sp, s0	*n* = 4; rostral sp, s0	*n* = 10; sp–s0	
*Pax3*	*n* = 4; ps, epiblast	*n* = 4; s0	*n* = 2; s0	*n* = 5; s0	*n* = 5; rostral sp, s0	*n* = 10; sp–s0	Prominent expression in the neural tube and in neural crest cells
*Pax7*	*n* = 4; ps	*n* = 4; s0	*n* = 5; s0	*n* = 4; s0	*n* = 4; rostral sp, s0	*n* = 9; sp–s0	Prominent expression in the neural tube and in neural crest cells
*Six1*	*n* = 2; head mesoderm	*n* = 2; s0	*n* = 7; s0	*n* = 2; s0	*n* = 5; rostral sp, s0	*n* = 10; rostral sp, s0	Expression in the HH5-10 head mesoderm
*Eya1*	*n* = 2; ps	*n* = 1; s0	*n* = 3; s0	*n* = 2; s0	*n* = 1; rostral sp, s0	*n* = 6; sp, s0	
*Myf5*	*n* = 3; –	*n* = 4; –	*n* = 7; s0–s1	*n* = 7; s0–s1	*n* = 8; s0–s1	*n* = 11; s0–1	
*MyoD*	*n* = 3; –	*n* = 3; –	*n* = 4; –	*n* = 8; s4/5	*n* = 6; s1/2	*n* = 7; s1/2	
*MyoG*	*n* = 1; –	*n* = 1; –	*n* = 1; –	*n* = 4; s9/10	*n* = 4; s6/7	*n* = 4; s5	
*Mrf4*	*n* = 2; –	*n* = 3; –	*n* = 2; –	*n* = 9; s9/10	*n* = 7; s8/9	*n* = 7; s6–8	Low overall expression levels
*Mef2a*	*n* = 5; ps	*n* = 2; –	*n* = 1; –	*n* = 7; s1	*n* = 8; s1; myotome s10	*n* = 1; s1	Low overall expression levels; HH7-16: widespread expression, strongest: cardiac precursors; HH16 onwards: upregulated in the mature myotome
*Mef2c*	*n* = 1; –	*n* = 8; weak signal in somites	*n* = 8; weak signal in s1–3	*n* = 5; s5–7	*n* = 4; s3/4	*n* = 3; s3	HH7-14: strongest signal in cardiac precursors
*Mef2d*	*n* = 1; –	*n* = 1; –	*n* = 1; –	*n* = 2; s6–8	*n* = 2; s7–9	*n* = 2; s4/5	Low overall expression levels, strongest: cardiac precursors
*Mef2b*	*n* = 1; –	*n* = 1; s1	*n* = 1; s1	*n* = 3; s1	*n* = 1; s1	*n* = 1; s1	HH7-14: widespread expression, strongest in ps and somites
*Cdh4*	*n* = 1; –	*n* = 2; –	*n* = 5; –	*n* = 6; s7–9	*n* = 2; s0,s1; then from s7/8 onwards	*n* = 10; s3/4	HH7-10: notochord; from HH13/14 onwards: intermediate mesoderm; low overall expression levels
*Desmin*	*n* = 1; –	*n* = 1; –	*n* = 1; –	*n* = 2; s10–12	*n* = 2; S11–13	*n* = 6; s7/8	Weak signal; HH13/14 onwards: prominent expression in heart; low overall expression levels
*Tnni1*	*n* = 1; –	*n* = 2; –	*n* = 1; –	*n* = 1; s9/10	*n* = 2; s5/6	*n* = 6; s4/5	HH7-10 onwards: cardiac precursors; heart
*Myh15*	*n* = 1; –	*n* = 2; –	*n* = 3; –	*n* = 3; s20/21	*n* = 3; s14/15	*n* = 2; s11/12	HH7-10 onwards: cardiac precursors; heart
*Myh7*	*n* = 1; –	*n* = 2; –	*n* = 3; –	*n* = 3; –	*n* = 3; s19/20	*n* = 2; s11–13	Low overall expression levels; HH7-10: precursors of cardiac inflow tract; HH13/14 onwards: heart
Sarcomeric myosins	*n* = 3; –	*n* = 1; –	*n* = 3; –	*n* = 2; s16/17	*n* = 4; s14–16	*n* = 4; s8–9	HH9/10 onwards: heart

### Expression of *Tbx6*

*Tbx6* expression commenced in the primitive streak before the stages shown here. At HH8 (Fig. 1A), the gene labelled the rostral primitive streak. Expression continued in the cells that leave the streak to settle as paraxial mesoderm; moreover, expression was found in the immature paraxial mesoderm prior to somite formation, known as segmental plate or pre-somitic mesoderm. Significantly, expression was still visible as an epithelial somite formed. At HH10 (Fig. 2A), a similar expression patterns was seen; in a strongly stained specimen, *Tbx6* expression was detectable up to the somite 2/3, labelling the medial-rostral edge of the somite most strongly. At HH14 (Fig. 3A) and HH16 (Fig. 4A), *Tbx6* labelled the paraxial mesodermal cells emerging from the tail bud. As before, expression continued in the segmental plate and the youngest somites. At HH19-20, mesoderm formation is almost complete. As few further cells are being added, the process of somite formation now consumes the segmental plate; hence the youngest somites are located close to the tail bud. *Tbx6* was expressed from the tailbud up to the youngest two somites (Figs 5A and 6A).

### Expression of *Paraxis*

*Paraxis* expression labelled the prospective somitic mesoderm as soon as HH4 (not shown). At HH8 (Fig. 1B), expression was found in the rostral segmental plate, continuing in somites as they segregated from the segmental plate. The same pattern was observed at HH10 (Fig. 2B), HH14 (Fig. 3B), HH16 (Fig. 4B) and HH19-20 (not shown). As somites matured, *Paraxis* expression became confined to the somitic dermomyotome and sclerotome; the strongest expression by far was found in the dorsomedial (epaxial) portion of the dermomyotome (Fig. 5B,Bi). Thus *Paraxis* expression partially overlapped with that of *Tbx6* but continued at high levels in myogenic precursor cells.

### Expression of *Pax3* and *Pax7*

#### Pax3

*Pax3* had a complex expression pattern, and at HH4-5 was expressed in the epiblast and along the primitive streak (not shown). This expression continued at HH8 (Fig. 1C) but, in addition, the lateral aspect of the condensing somites and the overlying edge of the neural plate (the neural folds) also expressed the gene. At HH10 (Fig. 2C), *Pax3* expression similarly encompassed the epiblast flanking the remnant of the primitive streak, the neural folds/dorsal neural tube and the condensing as well as well-formed somites. At HH14 (Fig. 3C), HH16 (Fig. 4C) and HH19-20 (Fig. 5C,Ci and not shown), *Pax3* expression was found in the tail bud, the dorsal neural tube, the lateral aspect of the condensing paraxial mesoderm and the somites. As somites matured, expression became restricted to the dermomyotome, with somewhat elevated levels in the dermomyotomal centre and very strong expression in the dorsomedial and ventrolateral lips (Fig. 5C,Ci). Thus, *Pax3* expression tightly overlapped with that of *Paraxis*, but areas of elevated expression levels were distinct.

#### Pax7

Expression of *Pax7* was very similar to its paralog *Pax3* (Figs 1–5D,Di). However, *Pax7* did not show prominent expression in the epiblast and tail bud but rather had elevated expression levels in the emigrating cranial neural crest cells. Somitic expression began in the rostral segmental plate as observed for *Pax3*. In the HH19-20 mature somite, expression was strongest in the dermomyotomal centre, occupying a dorsoventrally wider region than *Paraxis* (Fig. 5D,Di).

**Fig. 9:**
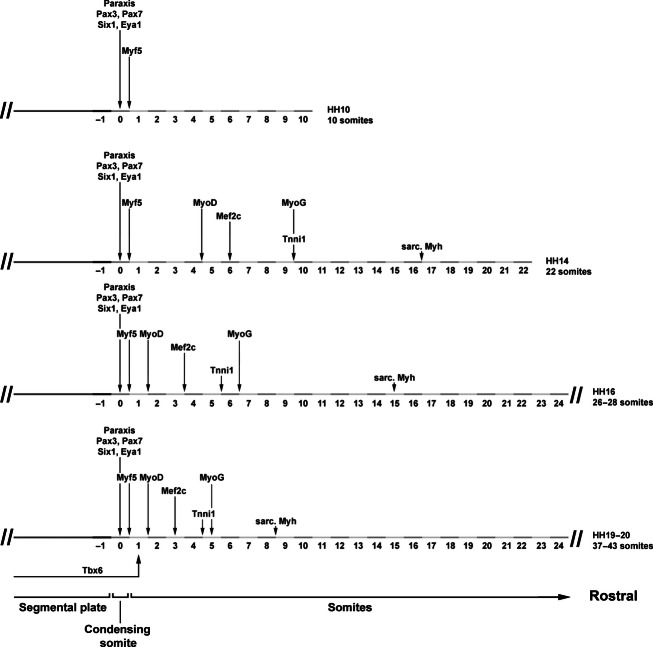
Summary. Progression of marker gene expression from HH10-HH19/20, focusing on the onset of the most strongly expressed genes, as their onset can be determined most precisely. At all times, the expression of *Tbx6*, of pre-myogenic genes and of *Myf5* overlaps. As development proceeds, the onset of markers associated with myogenic progression and terminal differentiation occurs earlier, indicating that the process accelerates in comparison with the progress of somite formation.

### Expression of *Six1*

*Six* genes evolved from an ancestral *Six1/2/sine oculis* gene, a *Six4/5* gene and a *Six3/6/optix* gene, with *Six4/5* and *optix*-related genes having arisen from an earlier, common ancestor (Kumar, 2009). In mouse and chicken, *Six1* and *Six4*/5 are co-expressed in the newly formed somite, the developing dermomyotome, eventually becoming confined to the dermomyotomal lips and the myotome; other *Six* genes don't show prominent somitic expression (Oliver et al. 1995; Esteve & Bovolenta, 1999; Heanue et al. 1999; Jean et al. 1999; Klesert et al. 2000; Grifone et al. 2005; Schubert & Lumsden, 2005). Single *Six1*, but not *Six4* or *5*, mutations cause somitic phenotypes, indicating that *Six1* is the most important player in myogenesis (Grifone et al. 2005). We therefore focused on *Six1* in this study. At HH4-5, *Six1* was expressed in the non-somitic head mesoderm and the pre-placodal ectoderm (not shown). At HH8 (Fig. 1E) and HH10 (Fig. 2E), this expression was accompanied by expression in the pre-chordal plate and the developing somites. Expression in the rostral segmental plate and somites was also evident at HH14 and HH16 (Figs 3E and 4E). In differentiating somites, strong *Six1* expression was maintained in the dermomyotome, thus overlapping with the expression of *Paraxis*, *Pax3* and *Pax7* (not shown). In contrast, in mature somites at HH19-20, the strongest expression was found in the dorsomedial and ventrolateral lips of the dermomyotome and the underlying myotome (Fig. 5E,Ei).

### Expression of *Eya1*

Eya proteins are protein tyrosine phosphatases which, among other roles, are able to convert Six proteins into strong transcriptional activators (Li et al. 2003; Tootle et al. 2003; reviewed in Tadjuidje & Hegde, 2013). Vertebrates have four *Eya* genes; in the mouse, the closely related *Eya1*, *2* genes and the more distantly related *Eya4* are co-expressed first in the dermomyotome and its dorsomedial and ventrolateral lips, and later in the myotome, and they have overlapping roles; *Eya3* shows weak somitic expression only but the protein cooperates with Ski and Six1 in the differentiation of C2C12 myoblasts (Xu et al. 1997; Borsani et al. 1999; Grifone et al. 2007; Zhang & Stavnezer, 2009). Expression of chicken *Eya2* has been described by Heanue et al. (1999) and matches that of the mouse, hence we focused on *Eya1*. The gene showed very similar expression to that of *Six1* at stages HH8, HH10, HH14 and HH16 of development (Figs 1F, 2F, 3F and 4F). In mature somites at HH19-20, the strongest expression was found in the dorsomedial and ventrolateral lips of the dermomyotome and in the myotome (Fig. 5F,Fi). This suggests that throughout somite development, Eya1 is available to Six1 to activate myogenic genes, and both are in the position continuously to drive myogenic differentiation in the myotome.

### Expression of *Mrf* genes

#### Myf5

*Myf5* has been portrayed as the earliest *Mrf* gene to be expressed. However, even though embryos at HH7-8 have one to four somites, Myf5 expression was not detectable (Fig. 1G). We first found a somewhat diffuse signal in condensing and newly formed somites at HH10, with somite 4/5 to somite 10 showing expression in their medial wall (Fig. 2G). This is the territory from which the cells building the primary myotome arise (Kahane et al. 1998b). At HH14 (Fig. 3G) and 16 (Fig. 4G), a similar pattern was observed. From somite 9 onwards, *Myf5* expression began to spread laterally, in tune with the establishment of this myotomal scaffold. In mature somites at HH19-20 (Figs 5G,Gi and 8A,Ai), *Myf5* labelled the sub-lip domain of both the dorsomedial as well as the ventrolateral lip, in tune with myogenic cell production from both lips (Denetclaw et al. 1997; Kahane et al. 1998a, 2007; Denetclaw & Ordahl, 2000). Moreover, prominent expression was seen throughout the myotome. However, the immediate sub-lip domains of the rostral and caudal lips that are also contributing to the myotome (Kahane et al. 1998a), did not express *Myf5* (Fig. 8A,Aii). Nevertheless, at a short distance from these lips, individual *Myf5*-positive cells were found (Fig. 8A, arrows), suggesting that after the entry into the myotome, cells derived from the rostrocaudal lips quickly activated *Myf5*.

#### MyoD

*MyoD* expression was not detected at stages HH4-10 of development (Figs 1H and 2H, and not shown). At HH14, the gene labelled the dorsomedial territory of somites 4/5 and older, as seen for *Myf5* (Fig. 3H); at H16, this expression was already seen in somites 1/2 (Fig. 4H). Expression expanded laterally as seen for *Myf5*, but lagging behind by one to two somites. At HH19-20, expression in mature somites was nearly indistinguishable from that of *Myf5* (Fig. 5H,Hi). However, the immediate dorsomedial and ventrolateral sub-lip domains were not stained, and expression appeared more punctuate than that of *Myf5*, suggesting that not all cells expressed *MyoD*.

#### MyoG

*MyoG* expression was first detected at HH13-14, i.e. in animals with a total count of 19–22 somites, commencing in somite 9/10 (Fig. 3I). As for *Myf5* and *MyoD*, expression spread laterally in older somites. Notably, at HH16, expression was already visible from somite 6/7 onwards (Fig. 4I), and at HH19-20, already the 5th youngest somite expressed the gene (Fig. 6B), suggesting that the progress of somite maturation speeds up as development progresses. In mature somites at HH19-20, *MyoG* was expressed throughout the myotome similar to *MyoD* (Fig. 5I,Ii); as for *MyoD*, not all cells appeared *MyoG*-positive.

#### Mrf4

*Mrf4* expression commenced in somites 9/10 at HH13-14, about concomitant with the expression of *MyoG* (Fig. 3J). At HH16, the first signal was seen in somites 8/9 (Fig. 4J) and at HH19-20, in somites 6–7 (not shown). Compared with the other *Mrf* genes, *Mrf4* expression levels were low. In mature somites, *Mrf4* expression was seen in the myotome; the sub-lip domains of the dermomytome were not stained (Fig. 5J,Ji). Expression was concentrated in the middle of the myotome, with the staining in the epaxial domain being stronger than in the hypaxial domain. Expression appeared even more punctuate than that of *MyoD* or *MyoG*, indicating that only a fraction of cells in the myotome expressed the gene.

### Expression of *Mef2* genes

Vertebrates have retained the four *Mef2* genes that were generated during their two rounds of genome duplication (Wu et al. 2011). Of these, *Mef2a* and *Mef2c* are thought to have arisen from one of the ancestral genes generated in the first duplication event, and the genes have remained rather similar. *Mef2b* and *Mef2d* are thought to stem from the other ancestral gene; however, *Mef2b* has evolved faster and is now rather divergent. The genes are displayed according to their similarity to *Mef2a*.

#### Mef2a

*Mef2a* was already expressed at HH4-5 in the primitive streak, albeit weakly (not shown). At stages HH7-8 and onwards, a low-level widespread staining was seen, with the strongest expression in the precursors of the primitive heart (Figs 1K and 2K; ht). The early heart, blood island and the notochord were prominent expression domains at HH14 (Fig. 3K and not shown) and from this stage onwards, the somites also showed *Mef2a* expression (Figs 3K and 4K). In mature somites at HH16 (Fig. 4K) and HH19-20 (Figs 5K,Ki), elevated expression was found in the myotome, in a pattern similar to that of *MyoG* and *Mrf4*.

#### Mef2c

*Mef2c* was co-expressed with *Mef2a* in the cardiac precursors of HH7-8 embryos and the primitive heart of HH10 embryos (Figs 1L and 2L; ht). At these stages, a diffuse, weak staining was also seen in the somites. At HH14, robust somitic expression was seen from somites 5/6 onwards, labelling the dorsomedial territory as seen for *MyoD* (Fig. 3L). At HH16, somites 3/4 were already *Mef2c*-positive (Fig. 4L), with expression spreading laterally as seen for *Mrf* genes. In mature somites at HH19-20, robust expression was seen in the myotome, with elevated expression in the myotomal centre (Fig. 5L,Li). Notably, *Mef2c* expression at that stage also strongly labelled domain beneath the rostral and caudal lips of the dermomytome (Fig. 8B,Bii, arrowheads).

#### Mef2d

*Mef2d* had low overall expression levels, and expression was just about detectable in cardiac precursors at HH7-8 and the primitive heart at HH10 (Figs 1M and 2M). At HH14, expression in the medial aspect of the somite was detectable from somite 6–8 onwards, and a similar range was displayed at HH16 (Figs 3M and 4M). At HH19-20, signals were found from somites 3/4 onwards. In the mature somites of the flank, expression was confined to the myotome.

#### Mef2b

*Mef2b* showed widespread expression in all germ layers, at HH7-8 most strongly labelling the primitive streak, the neural plate, the cardiac precursors and the somites (Fig. 1N). At HH10, the expression was similar; however, the somitic signal appeared weaker than that for the neural tube and primitive streak (Fig. 2N). Widespread expression was also seen at HH14, 16 and 19–20 (Figs 3N, 4N and 5N). Cross sections showed that the myotome expressed the gene similar to other *Mefs*, but the sclerotome was also positive (Fig. 5Ni).

### Expression of *Cadherin 4 (R-Cadherin, Cdh4)*

*Cdh4* has been shown to be expressed in the developing myotome, and its ability to support cell adhesion in epithelia suggests a role in myogenic cell alignment and cohesion (Inuzuka et al. 1991; Rosenberg et al. 1997). We found the first *Cdh4* expression in the developing notochord at HH7-8 and 9–10 (Figs 1O and 2O). At HH14 (Fig. 3O), the gene showed a complex expression pattern, encompassing the notochord and the intermediate mesoderm-derived nephric duct. Weak expression was seen in the condensing somite and the first one to three newly formed somites. More robust expression, however, was found in the medial territory of somites 7–9. In further rostral somites, the signal spread laterally, concomitant with the developing myotome as seen for *Mrf* genes. Notably, nine to 10 somites rostral to the somite expressing the gene first, a new expression domain emerged in the lateral lip and sub-lip domain of the dermomyotome. At HH16 (Fig. 4O) and HH19-20 (Fig. 5O and not shown), a similar pattern was observed. Cross sections of HH19-20 flank somites confirmed *Cdh4* expression in the myotome and throughout the ventrolateral dermomyotomal lip (Fig. 5Oi).

### Expression of muscle structural genes

Desmin, Tnni1 (Troponin I 1), Myh15 (Myosin Heavy Chain 15 or ventricular Myosin Heavy Chain) and Myh7 (Myosin Heavy Chain, slow/cardiac or atrial Myosin Heavy Chain) are components of the functional sarcomere and have been reported to be expressed in the early embryo (http://geisha.arizona.edu). We therefore included these markers in our analysis. To monitor the availability of Myosins independent of individual contributing genes, we used the pan-sarcomeric Myosin MF20 antibody. To evaluate the levels of protein production, we performed whole mount antibody stainings on HH21 embryos with antibodies known (Desmin) or predicted (Tnni1, Myh17, Myh7) to recognise the avian proteins.

#### Desmin

*Desmin* mRNA was first detected at HH13-14, labelling somites 10–12 and older (Fig. 3P). At HH16, a similar expression was found (Fig. 4P). At HH19-20, expression was detected already in somites 7/8 (not shown). Expression was confined to the centre of the myotome which contains the myonuclei (Fig. 5P, Pi). Overall, somitic *Desmin* transcription was low; signals in the heart were more prominent. Notably, Desmin protein was more readily detectable (Fig. 7H), suggesting that the production rate or half-life of the protein is higher than that of the mRNA. In contrast to the transcript, the protein was evenly distributed along the myotube, suggesting an active distribution mechanism.

#### Tnni1

*Tnni1* mRNA was expressed weakly in the rostral primitive streak and the lateral mesoderm at HH4-5 (not shown). Throughout the stages analysed here, cardiac precursors and heart were the most prominent expression domains (see Figs 1Q and 2Q; ht). At HH14, somites 9/10 expressed the gene (Fig. 3Q), at HH16 expression started already in somites 5/6 (Fig. 4Q) and at HH19-20 in somites 4/5 (Fig. 6C). Throughout, expression was confined to the myotome (Fig. 5Q,Qi). *Tnni1* transcripts were readily detectable, as was the Tnni1 protein (Fig. 7I).

#### Myh15

*Myh15* expression was visible from HH7-8 onwards, labelling the cardiac precursors and subsequently the heart (Figs 1R and 2R; ht; Bisaha & Bader, 1991). Myogenic expression was first detected at HH14 in somites 20/21, i.e. the oldest two somites (Fig. 3R). At HH16 expression was seen earlier, namely in somites 14/15, at this stage starting in the myotomal centre (Figs 4R and 7A). At HH19/20, expression appeared as early as somites 11/12. Expression was confined to the developing myotubes, with the strongest expression at their rostral and caudal extremities (Figs 5R,Ri and 7B–D, arrows). In comparison with the mRNA, protein detection was less robust. However, at HH21, the protein was also enriched along the rostral and caudal edges of the myotome (Fig. 7J).

#### Myh7

As reported by Oana et al. (1998) *Myh7* expression levels were low, lower than those of *Myh15*. At HH7-8 the gene was expressed in cardiac precursors, and from HH9/10 onwards, expression was found in the atrium of the heart (Figs 9S; ht). At HH16, somites 19/20 expressed the gene (Fig. 4S), and at HH19-20, expression was visible from somites 11–13 onwards (Fig. 5S). Expression was confined to the developing myotubes, most strongly labelling their centre (Fig. 7E–G, arrow). Myh7 protein was difficult to detect, but also appeared enriched in the myotomal centre (Fig. 7K).

### Pan-sarcomeric Myosin detection

The MF20 antibody recognises the rod-like tail of all sarcomeric Myosins and therefore is a readout for cardiac and skeletal muscle terminal differentiation independent of the individual contributing *Myosin* gene (Bader et al. 1982). Initially, the antibody only detected the developing heart (Fig. 2T, ht). At HH14, somites 16/17 and older were stained (Fig. 3T), at HH16 staining was already found in somites 14–16 (Fig. 4T) and at HH19/20 in somites 8–9 (Fig. 6D). Expression was confined to myotubes (Figs 5T,Ti and 7L).

### Comparative analysis of *Myf5*, *Mef2c*, *Follistatin* and *Pitx3* along the four dermomyotomal lips

*Myf5* and pre-myogenic genes shown here displayed overlapping expression first in the dorsomedial and then in the ventrolateral lip of the dermomytome. Expression of *MyoD*, *MyoG*, the *Mef2* genes and *Cdh4* expression overlapped with that of *Myf5* in the dorsomedial and ventrolateral sub-lip domains. In contrast, *Mef2c* eventually also displayed expression along the rostrocaudal dermomyotomal lips (compare Fig. 8A,Ai,Aii,B,Bi,Bii). We screened our embryo collection for additional markers labelling this territory. We found that the gene encoding the transforming growth factor beta (TGFb) inhibitor Follistatin was expressed in condensing and newly formed somites from HH6 onwards (Bothe et al. 2011). Expression continued in the dermomyotome, with upregulated expression along all four dermomyotomal lips (not shown). In mature somites of the HH19/20 flank, expression was visible in these lips as well as in the myotome (Fig. 8C,Ci,Cii). *Pitx3* expression initially labelled the lens of the eye. At HH16 (not shown) and 19/20 (Fig. 8D,Di,Dii) expression was found in the mature somitic myotomes. Notably, expression was strongest along the rostrocaudal edges of the myotome, with the most prominent expression found in the lateral aspect of the caudal sub-lip domain.

## Concluding remarks

The aim of this study was to provide, in the avian model for somitic myogenesis, a side-by-side analysis of the key markers associated with the progression from an immature state of the paraxial mesoderm to myogenic commitment and, eventually, to myogenic differentiation. Our study also provided novel insight into the process of skeletal muscle formation.

### All cells in the somite have a history of pre-myogenic gene expression

Our study shows that the immature paraxial mesoderm invariably expresses *Tbx6*. When the tissue condenses and epithelial somites form, *Tbx6* signals fade away and expression of the pre-myogenic genes (*Paraxis, Pax3, Pax7, Six1, Eya1*) begins. Notably, all pre-myogenic genes first label the entire developing somite before expression becomes confined to the dermomyotome; for *Six1* and *Eya1*, expression eventually becomes further restricted to the dorsomedial and ventrolateral lips of the dermomyotome but continues when cells enter the myotome. Thus, in contrast to anamniotes (Hinits et al. 2009; Della Gaspera et al. 2012), myogenic cells in the chicken somite have a history of pre-myogenic marker gene expression.

### *Myf5* is the first gene to indicate myogenic commitment

*Mrf* genes are thought to drive cells into myogenesis, with *Myf5* and *MyoD* playing similar roles in the still mitosis-competent myoblast (reviewed in Aziz et al. 2010; Fong & Tapscott, 2013). We found, however, that *Myf5* was always the first *Mrf* to be expressed, showing a diffuse expression in the epithelialising somite, and then a robust expression in the medial wall of a newly formed somite, the dorsomedial lip of the dermomyotome and the expanding myotome; later, this expression was mirrored in the ventrolateral aspect of the somite. *MyoD* was activated after *Myf5*, and expression was found in the dorsomedial and ventrolateral sub-lip domains of the dermomyotome, and not in the dermomyotomal lips themselves. This suggests that *Myf5* is a marker for myogenic commitment, whereas *MyoD* indicates cells ready to enter differentiation. Significantly, no sign of myogenic differentiation was ever seen at the start of *Myf5* expression. Thus, *Myf5* may not be as capable to drive myogenesis *in vivo* as *in vitro*. Alternatively, the continued expression of genes associated with an immature state such as *Tbx6* may be a contributing factor. Furthermore, it was shown that in quiescent satellite cells, the *Myf5* mRNA is held together with miR31 in mRNP granules, preventing *Myf5* translation (Crist et al. 2012), and this mechanisms may also operate in the embryo. Interestingly, an expression profile similar to that of *Myf5* has recently been shown for avian *Rgm* genes (Jorge et al. 2012), and it will be interesting to learn about the functional relationship of these genes.

### In contrast to the mouse, *Mrf4* (*Myf6*) is the last *Mrf* to be expressed

During all stages investigated here, *Myf5* expression was always followed by that of *MyoD*, which was followed by the expression of *MyoG* and *Mrf4*; an early onset of *Mrf4* expression as shown in the mouse (Summerbell et al. 2002) has not been observed. It is not clear whether the murine expression patterns are typical for all mammals but the avian sequence of *Mrf* expression is akin to that shown for *Xenopus* and zebrafish, suggesting that this is the basic configuration for jawed vertebrates. *MyoG* is known to promote cell cycle exit and terminal differentiation (reviewed in Aziz et al. 2010; Fong & Tapscott, 2013), yet *MyoD*, *MyoG* and *Mrf* were eventually all co-expressed in the dorsomedial and ventrolateral sub-lip domains of the myotome. This suggests that cells entering the myotome via these lips withdraw from cell cycle and begin their differentiation within this compartment before being displaced to a position away from the lips by the next cells entering from the dermomyotome.

### Mef2c is the likely partner for Mrf proteins in myogenesis

*Mrf* genes are key regulators of myogenic commitment and differentiation. Yet they need to interact with Six and Mef2 proteins to be able to activate target gene transcription (reviewed in Aziz et al. 2010; Fong & Tapscott, 2013). Our analysis suggests that Six and Eya gene products are available to Mrf all the time, as the genes were expressed in epithelialising somites, the early dermomyotome, and then the dorsomedial and ventrolateral dermomyotomal lips as well as the myotome. Of the *Mef2* genes, however, only *Mef2c* showed robust expression in the developing somites, suggesting that *Mef2c* is the most likely Mrf partner. *Mef2c* expression followed that of *MyoD*, and this may contribute to the fact that terminal differentiation does not occur prior to the onset of *MyoD* expression.

### Differentiation catches up with somitogenesis

At a given state, the precise onset of marker gene expression varied slightly, possibly because embryos were in a different phase of segmentation and epithelial somite formation. Moreover, for weakly expressed genes, the duration of the staining reaction (up to 2 weeks) led to somewhat divergent results. However, the sequence of marker gene expression was similar at all stages investigated (shown for the robustly expressed genes in Fig. 9). At early stages of development, markers indicating entry into differentiation were not yet expressed (HH10) or were expressed a distance to the segmental plate (HH14), but this distance decreased as development proceeded (HH16, HH19/20), indicating that the process of differentiation catches up with the process of somitogenesis, and may contribute to the eventual consumption of immature cells in the tail bud of the embryo. It has been suggested that the decline of Wnt and Fgf signalling in the tail bud, combined with the expression of Raldh2 that leads to elevated retinoic acid levels, controls the cessation of somite formation and body elongation (Tenin et al. 2010; Rashid et al. 2014). However, these changes occur after the time period considered here, indicating that additional molecular players contribute to the acceleration of somite differentiation.

### Distinct combinations of marker genes label cells from the dorsomedial, ventrolateral and rostrocaudal dermomyotomal lips

*Myf5* expression was tightly associated with cells in the dorsomedial and ventrolateral lips of the dermomyotome and the associated sub-lip domains, which account for the incremental growth of the myotome (Kahane et al. 1998a, 2007; Denetclaw & Ordahl, 2000; Pu et al. 2013). However, the rostrocaudal lips are a further, important source of myogenic cells and are thought to drive the expansion of the myotomal centre (Kahane et al. 1998a). Although *Mef2c* and *Follistatin* expression labelled cells emerging from all dermomyotomal lips, this was not the case for *Mrf*. Moreover, the rostrocaudal lips but not the dorsomedial and ventrolateral lips expressed *Pitx3*, suggesting that distinct cascades control myogenesis from the four lips. However, *Myf5* was expressed in cells at a short distance from the rostrocaudal lips, suggesting that, eventually, all cells express *Mrf* and programs converge.

### Gene products are differentially distributed along the rostrocaudal length of the myotube

The contractile protein complexes of skeletal muscle (myofibrils) are built from repetitive protein units, the sarcomeres, and sarcomeres have a stereotype arrangement of proteins (Alberts et al. 1983). It is therefore interesting to note that the gene products of the *Myh15* and *Myh7* genes, while initially mainly labelling the myotomal centre, became differentially distributed as the myotomes matured, with *Myh15* mRNA and protein being enriched at the rostrocaudal extremes and *Myh7* gene products in the centre. Given that *Myh7* expression lags behind that of *Myh15*, it is possible that *Myh7* gene products may eventually become similarly redistributed as the products of *Myh15*. However, it is also possible that the contractile properties along the length of a myotube are different. Reports on Fgf signalling molecules showed that many are specifically expressed in the myotomal centre and control the release of the embryonic muscle stem cells from the overlying dermomyotome (Karabagli et al. 2002; Delfini et al. 2009). It will be interesting to explore in the future whether and how both processes are linked.

## Supporting Information

**Table S1.** Probes used for whole mount *in situ* hybridisation. **Table S2.** Antibodies used in this study.

## References

[b1] Abmayr SM, Pavlath GK (2012). Myoblast fusion: lessons from flies and mice. Development.

[b2] Ahmed MU, Cheng L, Dietrich S (2006). Establishment of the epaxial-hypaxial boundary in the avian myotome. Dev Dyn.

[b3] Alberts B, Bray D, Lewis J (1983). Molecular Biology of the Cell.

[b5] Alvares LE, Schubert FR, Thorpe C (2003). Intrinsic, Hox-dependent cues determine the fate of skeletal muscle precursors. Dev Cell.

[b6] Atchley WR, Fitch WM (1997). A natural classification of the basic helix-loop-helix class of transcription factors. Proc Natl Acad Sci U S A.

[b7] Aziz A, Liu QC, Dilworth FJ (2010). Regulating a master regulator: establishing tissue-specific gene expression in skeletal muscle. Epigenetics.

[b8] Bader D, Masaki T, Fischman DA (1982). Immunochemical analysis of myosin heavy chain during avian myogenesis in vivo and in vitro. J Cell Biol.

[b9] Ben-Yair R, Kalcheim C (2005). Lineage analysis of the avian dermomyotome sheet reveals the existence of single cells with both dermal and muscle progenitor fates. Development.

[b10] Bergstrom DA, Tapscott SJ (2001). Molecular distinction between specification and differentiation in the myogenic basic helix-loop-helix transcription factor family. Mol Cell Biol.

[b11] Bisaha JG, Bader D (1991). Identification and characterization of a ventricular-specific avian myosin heavy chain, VMHC1: expression in differentiating cardiac and skeletal muscle. Dev Biol.

[b12] Black BL, Molkentin JD, Olson EN (1998). Multiple roles for the MyoD basic region in transmission of transcriptional activation signals and interaction with MEF2. Mol Cell Biol.

[b13] Borsani G, DeGrandi A, Ballabio A (1999). EYA4, a novel vertebrate gene related to Drosophila eyes absent. Hum Mol Genet.

[b14] Bothe I, Tenin G, Oseni A (2011). Dynamic control of head mesoderm patterning. Development.

[b15] Braun T, Bober E, Buschhausen-Denker G (1989a). Differential expression of myogenic determination genes in muscle cells: possible autoactivation by the Myf gene products. EMBO J.

[b16] Braun T, Buschhausen-Denker G, Bober E (1989b). A novel human muscle factor related to but distinct from MyoD1 induces myogenic conversion in 10T1/2 fibroblasts. EMBO J.

[b17] Bryson-Richardson RJ, Currie PD (2008). The genetics of vertebrate myogenesis. Nat Rev Genet.

[b18] Buckingham M, Vincent SD (2009). Distinct and dynamic myogenic populations in the vertebrate embryo. Curr Opin Genet Dev.

[b19] Burgess R, Rawis A, Brown D (1996). Requirement of the *paraxis* gene for somite formation and muscoloskeletal patterning. Nature.

[b20] Burt DW (2007). Emergence of the chicken as a model organism: implications for agriculture and biology. Poult Sci.

[b21] Cao Y, Kumar RM, Penn BH (2006). Global and gene-specific analyses show distinct roles for Myod and Myog at a common set of promoters. EMBO J.

[b22] Cao Y, Yao Z, Sarkar D (2010). Genome-wide MyoD binding in skeletal muscle cells: a potential for broad cellular reprogramming. Dev Cell.

[b23] Chapman DL, Papaioannou VE (1998). Three neural tubes in mouse embryos with mutations in the T-box gene Tbx6. Nature.

[b24] Chapman DL, Cooper-Morgan A, Harrelson Z (2003). Critical role for Tbx6 in mesoderm specification in the mouse embryo. Mech Dev.

[b26] Clack JA (2002). Gaining Ground. The Origin and Evolution of Tetrapods.

[b27] Cogburn LA, Porter TE, Duclos MJ (2007). Functional genomics of the chicken–a model organism. Poult Sci.

[b777] Crist CG, Montarras D, Buckingham M (2012). Muscle satellite cells are primed for myogenesis but maintain quiescence with sequestration of Myf5 mRNA targeted by microRNA-31 in mRNP granules. Cell Stem Cell.

[b28] Collins CA, Gnocchi VF, White RB (2009). Integrated functions of Pax3 and Pax7 in the regulation of proliferation, cell size and myogenic differentiation. PLoS One.

[b29] Delfini MC, De La Celle M, Gros J (2009). The timing of emergence of muscle progenitors is controlled by an FGF/ERK/SNAIL1 pathway. Dev Biol.

[b30] Della Gaspera B, Armand AS, Sequeira I (2012). Myogenic waves and myogenic programs during *Xenopus* embryonic myogenesis. Dev Dyn.

[b31] Denetclaw WF, Ordahl CP (2000). The growth of the dermomyotome and formation of early myotome lineages in thoracolumbar somites of chicken embryos. Development.

[b32] Denetclaw WF, Christ B, Ordahl CP (1997). Location and growth of epaxial myotome precursor cells. Development.

[b33] Dietrich S, Schubert FR, Lumsden A (1997). Control of dorsoventral pattern in the chick paraxial mesoderm. Development.

[b34] Dietrich S, Schubert FR, Healy C (1998). Specification of the hypaxial musculature. Development.

[b35] Dietrich S, Abou-Rebyeh F, Brohmann H (1999). The role of SF/HGF and c-Met in the development of skeletal muscle. Development.

[b36] Edmondson DG, Olson EN (1989). A gene with homology to the myc similarity region of MyoD1 is expressed during myogenesis and is sufficient to activate the muscle differentiation program. Genes Dev.

[b37] Edmondson DG, Cheng TC, Cserjesi P (1992). Analysis of the myogenin promoter reveals an indirect pathway for positive autoregulation mediated by the muscle-specific enhancer factor MEF-2. Mol Cell Biol.

[b38] Esteve P, Bovolenta P (1999). cSix4, a member of the six gene family of transcription factors, is expressed during placode and somite development. Mech Dev.

[b39] Fong AP, Tapscott SJ (2013). Skeletal muscle programming and re-programming. Curr Opin Genet Dev.

[b40] Gianakopoulos PJ, Mehta V, Voronova A (2011). MyoD directly up-regulates premyogenic mesoderm factors during induction of skeletal myogenesis in stem cells. J Biol Chem.

[b41] Gilbert SF (2000). Developmental Biology.

[b44] Grifone R, Laclef C, Spitz F (2004). Six1 and Eya1 expression can reprogram adult muscle from the slow-twitch phenotype into the fast-twitch phenotype. Mol Cell Biol.

[b45] Grifone R, Demignon J, Houbron C (2005). Six1 and Six4 homeoproteins are required for Pax3 and Mrf expression during myogenesis in the mouse embryo. Development.

[b46] Grifone R, Demignon J, Giordani J (2007). Eya1 and Eya2 proteins are required for hypaxial somitic myogenesis in the mouse embryo. Dev Biol.

[b47] Gros J, Manceau M, Thome V (2005). A common somitic origin for embryonic muscle progenitors and satellite cells. Nature.

[b48] Hamburger V, Hamilton HL (1951). A series of normal stages in the development of the chick embryo. J Morphol.

[b49] Heanue TA, Reshef R, Davis RJ (1999). Synergistic regulation of vertebrate muscle development by Dach2, Eya2, and Six1, homologs of genes required for *Drosophila* eye formation. Genes Dev.

[b50] Hindi SM, Tajrishi MM, Kumar A (2013). Signaling mechanisms in mammalian myoblast fusion. Sci Signal.

[b51] Hinits Y, Osborn DP, Hughes SM (2009). Differential requirements for myogenic regulatory factors distinguish medial and lateral somitic, cranial and fin muscle fibre populations. Development.

[b52] Holland LZ, Schubert M, Kozmik Z (1999). AmphiPax3/7, an amphioxus paired box gene: insights into chordate myogenesis, neurogenesis, and the possible evolutionary precursor of definitive vertebrate neural crest. Evol Dev.

[b53] Hubaud A, Pourquie O (2014). Signalling dynamics in vertebrate segmentation. Nat Rev Mol Cell Biol.

[b54] Hutcheson DA, Zhao J, Merrell A (2009). Embryonic and fetal limb myogenic cells are derived from developmentally distinct progenitors and have different requirements for beta-catenin. Genes Dev.

[b55] Inuzuka H, Redies C, Takeichi M (1991). Differential expression of R- and N-cadherin in neural and mesodermal tissues during early chicken development. Development.

[b56] Jean D, Bernier G, Gruss P (1999). Six6 (Optx2) is a novel murine Six3-related homeobox gene that demarcates the presumptive pituitary/hypothalamic axis and the ventral optic stalk. Mech Dev.

[b57] Jorge EC, Ahmed MU, Bothe I (2012). RGMa and RGMb expression pattern during chicken development suggest unexpected roles for these repulsive guidance molecules in notochord formation, somitogenesis, and myogenesis. Dev Dyn.

[b58] Kahane N, Cinnamon Y, Kalcheim C (1998a). The cellular mechanism by which the dermomyotome contributes to the second wave of myotome development. Development.

[b59] Kahane N, Cinnamon Y, Kalcheim C (1998b). The origin and fate of pioneer myotomal cells in the avian embryo. Mech Dev.

[b60] Kahane N, Cinnamon Y, Bachelet I (2001). The third wave of myotome colonization by mitotically competent progenitors: regulating the balance between differentiation and proliferation during muscle development. Development.

[b61] Kahane N, Ben-Yair R, Kalcheim C (2007). Medial pioneer fibers pattern the morphogenesis of early myoblasts derived from the lateral somite. Dev Biol.

[b62] Karabagli H, Karabagli P, Ladher RK (2002). Survey of fibroblast growth factor expression during chick organogenesis. Anat Rec.

[b63] Kassar-Duchossoy L, Gayraud-Morel B, Gomes D (2004). Mrf4 determines skeletal muscle identity in Myf5: Myod double-mutant mice. Nature.

[b64] Klesert TR, Cho DH, Clark JI (2000). Mice deficient in Six5 develop cataracts: implications for myotonic dystrophy. Nat Genet.

[b65] Kumar JP (2009). The sine oculis homeobox (SIX) family of transcription factors as regulators of development and disease. Cell Mol Life Sci.

[b66] Lepper C, Conway SJ, Fan CM (2009). Adult satellite cells and embryonic muscle progenitors have distinct genetic requirements. Nature.

[b67] Li X, Oghi KA, Zhang J (2003). Eya protein phosphatase activity regulates Six1-Dach-Eya transcriptional effects in mammalian organogenesis. Nature.

[b68] Linker C, Lesbros C, Gros J (2005). β-Catenin-dependent Wnt signalling controls the epithelial organisation of somites through the activation of paraxis. Development.

[b69] Lours C, Dietrich S (2005). The dissociation of the Fgf-feedback loop controls the limbless state of the neck. Development.

[b70] von Maltzahn J, Jones AE, Parks RJ (2013). Pax7 is critical for the normal function of satellite cells in adult skeletal muscle. Proc Natl Acad Sci U S A.

[b71] Mansouri A, Gruss P (1998). Pax3 and Pax7 are expressed in commissural neurons and restrict ventral neuronal identity in the spinal cord. Mech Dev.

[b72] McGrew MJ, Pourquie O (1998). Somitogenesis: segmenting a vertebrate. Curr Opin Genet Dev.

[b73] Miner JH, Wold B (1990). Herculin, a fourth member of the MyoD family of myogenic regulatory genes. Proc Natl Acad Sci U S A.

[b74] Molkentin JD, Black BL, Martin JF (1995). Cooperative activation of muscle gene expression by MEF2 and myogenic bHLH proteins. Cell.

[b75] Mootoosamy RC, Dietrich S (2002). Distinct regulatory cascades for head and trunk myogenesis. Development.

[b76] Naya FJ, Olson E (1999). MEF2: a transcriptional target for signaling pathways controlling skeletal muscle growth and differentiation. Curr Opin Cell Biol.

[b77] Oana S, Machida S, Hiratsuka E (1998). The complete sequence and expression patterns of the atrial myosin heavy chain in the developing chick. Biol Cell.

[b78] Oliver G, Wehr R, Jenkins NA (1995). Homeobox genes and connective tissue patterning. Development.

[b79] Penn BH, Bergstrom DA, Dilworth FJ (2004). A MyoD-generated feed-forward circuit temporally patterns gene expression during skeletal muscle differentiation. Genes Dev.

[b80] Pu Q, Abduelmula A, Masyuk M (2013). The dermomyotome ventrolateral lip is essential for the hypaxial myotome formation. BMC Dev Biol.

[b81] Rashid DJ, Chapman SC, Larsson HC (2014). From dinosaurs to birds: a tail of evolution. Evodevo.

[b82] Rawls A, Morris JH, Rudnicki MA (1995). Myogenin's functions do not overlap with those of MyoD or Myf-5 during mouse embryogenesis. Dev Biol.

[b83] Relaix F, Zammit PS (2012). Satellite cells are essential for skeletal muscle regeneration: the cell on the edge returns centre stage. Development.

[b84] Relaix F, Rocancourt D, Mansouri A (2004). Divergent functions of murine Pax3 and Pax7 in limb muscle development. Genes Dev.

[b85] Relaix F, Rocancourt D, Mansouri A (2005). A Pax3/Pax7-dependent population of skeletal muscle progenitor cells. Nature.

[b86] Relaix F, Montarras D, Zaffran S (2006). Pax3 and Pax7 have distinct and overlapping functions in adult muscle progenitor cells. J Cell Biol.

[b87] Relaix F, Demignon J, Laclef C (2013). Six homeoproteins directly activate Myod expression in the gene regulatory networks that control early myogenesis. PLoS Genet.

[b88] Rosenberg P, Esni F, Sjodin A (1997). A potential role of R-cadherin in striated muscle formation. Dev Biol.

[b89] Schubert FR, Lumsden A (2005). Transcriptional control of early tract formation in the embryonic chick midbrain. Development.

[b90] Schubert FR, Tremblay P, Mansouri A (2001). Early mesodermal phenotypes in splotch suggest a role for Pax3 in the formation of epithelial somites. Dev Dyn.

[b91] Seale P, Sabourin LA, Girgis-Gabardo A (2000). Pax7 is required for the specification of myogenic satellite cells. Cell.

[b92] Somorjai IM, Somorjai RL, Garcia-Fernandez J (2012). Vertebrate-like regeneration in the invertebrate chordate amphioxus. Proc Natl Acad Sci U S A.

[b93] Šošic D, Brand-Saberi B, Schmidt C (1997). Regulation of paraxis expression and somite formation by ectoderm- and neural tube-derived signals. Dev Biol.

[b94] Spitz F, Demignon J, Porteu A (1998). Expression of myogenin during embryogenesis is controlled by Six/sine oculis homeoproteins through a conserved MEF3 binding site. Proc Natl Acad Sci U S A.

[b95] Summerbell D, Halai C, Rigby PW (2002). Expression of the myogenic regulatory factor Mrf4 precedes or is contemporaneous with that of Myf5 in the somitic bud. Mech Dev.

[b96] Tadjuidje E, Hegde RS (2013). The eyes absent proteins in development and disease. Cell Mol Life Sci.

[b97] Tajbakhsh S, Rocancourt D, Cossu G (1997). Redefining the genetic hierarchies controlling skeletal myogenesis: Pax-3 and Myf-5 act upstream of MyoD. Cell.

[b98] Tenin G, Wright D, Ferjentsik Z (2010). The chick somitogenesis oscillator is arrested before all paraxial mesoderm is segmented into somites. BMC Dev Biol.

[b99] Tootle TL, Silver SJ, Davies EL (2003). The transcription factor Eyes absent is a protein tyrosine phosphatase. Nature.

[b100] Tremblay P, Dietrich S, Meriskay M (1998). A crucial role for *Pax3* in the development of the hypaxial musculature and the long-range migration of muscle precursors. Dev Biol.

[b101] Wang Y, Jaenisch R (1997). Myogenin can substitute for Myf5 in promoting myogenesis but less efficiently. Development.

[b102] Weintraub H, Tapscott SJ, Davis RL (1989). Activation of muscle-specific genes in pigment, nerve, fat, liver, and fibroblast cell lines by forced expression of MyoD. Proc Natl Acad Sci U S A.

[b103] Wilson-Rawls J, Hurt CR, Parsons SM (1999). Differential regulation of epaxial and hypaxial muscle development by paraxis. Development.

[b104] Windner SE, Bird NC, Patterson SE (2012). Fss/Tbx6 is required for central dermomyotome cell fate in zebrafish. Biol Open.

[b105] Wu W, de Folter S, Shen X (2011). Vertebrate paralogous MEF2 genes: origin, conservation, and evolution. PLoS One.

[b106] Xu PX, Woo I, Her H (1997). Mouse Eya homologues of the *Drosophila* eyes absent gene require Pax6 for expression in lens and nasal placode. Development.

[b107] Zhang H, Stavnezer E (2009). Ski regulates muscle terminal differentiation by transcriptional activation of Myog in a complex with Six1 and Eya3. J Biol Chem.

[b108] Zheng X, Wang Y, Yao Q (2009). A genome-wide survey on basic helix-loop-helix transcription factors in rat and mouse. Mamm Genome.

