# Supplementary material for: Time course and side-by-side analysis of mesodermal, pre-myogenic, myogenic and differentiated cell markers in the chicken model for skeletal muscle formation
Source: J Anat. 2015 Aug 17;227(3):361–82. doi: 10.1111/joa.12353 (PMC4560570; doi:10.1111/joa.12353)
Supplement: Supplementary file 1 [file joa0227-0361-sd1.docx]

**Supplementary Material: Table 1**

Probes used for whole mount in situ hybridisation. Clonings into the vectors pMK-RQ and pMA-T were carried out by Life Technologies, Germany.

| Gene | Source | Fragment size (base pairs) |
| --- | --- | --- |
| *Tbx6* | RT- PCR fragment obtained from HH13-17 cDNA using the primers  F: 5’- CCTGCTGGTGAGCAGGGAG-3’  RT7: 5’-TAATACGACTCACTATAGGGAGA-  CTGCATGATCCACAGCCGCG-3’  The fragment was verified by sequencing. | 597 |
| *Paraxis* | gift from E. Olson(Šošic et al., 1997) | 717 |
| *Pax3* | gift from P. Gruss (Goulding et al., 1993) | 660 |
| *Pax7* | gift from P. Gruss (Goulding et al., 1993) | 582 |
| *Six1* | gift from C. Tabin (Heanue et al., 1999) | 700 |
| *Eya1* | gift from A. Streit (Christophorou et al., 2009) | 1000 |
| *Myf5* | open reading frame, synthesised and cloned into pMK-RQ | 785 |
| *MyoD* | open reading frame, synthesised and cloned into pMK-RQ | 909 |
| *MyoG* | open reading frame, synthesised and cloned into pMK-RQ | 694 |
| *Mrf4* | open reading frame, synthesised and cloned into pMK-RQ | 738 |
| *Mef2a*-probe 1 | EST clone WTSIp6101M23667Q | 730 |
| *Mef2a*-probe 2 | open reading frame, synthesised and cloned into pMK-RQ | 1647 |
| *Mef2c* probe 1 | gift from T. Schultheiss (Alsan and Schultheiss, 2002) | 500 |
| *Mef2c* probe 2 | open reading frame, synthesised and cloned into pMK-RQ | 1431 |
| *Mef2d* | RT- PCR fragment obtained from HH13-17 cDNA using the primers  F: 5'-CGTGACCAACCAGAACACAC-3'  RT7: 5'-TAATACGACTCACTATAGGGAGA-GAGTGAGTCGCTTGGGAGAC-3'  The fragment was verified by sequencing. | 450 |
| *Mef2b* | RT- PCR fragment obtained from HH13-17 cDNA using the primers  F: 5’-AATGAGGGCATGGATCTGAC-3’  R: 5’-TAATACGACTCACTATAGGGAGA-GAAACCAGTGAAGGCTGTGG -3’  The fragment was verified by sequencing. | 414 |
| *Cdh4 = Cadherin 4 = R-Cadherin* | gift from C. Redies (unpublished PCR fragment) | 900 |
| *Des = Desmin* | 3’ half of the open reading frame, synthesised and cloned into pMK-RQ | 747 |
| *Troponin I 1 (Tnni 1)* | RT- PCR fragment obtained from HH13-17 cDNA using the primers  F: 5’-AGCAGCTCCCAGGAGATCAG-3’;  RT7: 5’-TAATACGACTCACTATAGGGAGA-CATGCAGCTGCATGGGCAC-3’  The fragment was verified by sequencing. | 921 |
| *Myh15* | position 1168-2388 of the open reading frame, synthesised and cloned into pMA-T | 1224 |
| *Myh7* | position 2173-3138 of the open reading frame, synthesised and cloned into pMA-T | 966 |
| *Follistatin* | gift from A. Graham (Graham and Lumsden, 1996) | 800 |
| *Pitx3* | gift from T. Ogura (unpublished PCR fragment, 3’ half of open reading frame) | 1000 |

**Supplementary Material: Table 2**

Antibodies used in this study.

| **primary antibodies** | **supplier** | **antigenic determinant** | **type** | **antigen retrieval** | **dilution** |
| --- | --- | --- | --- | --- | --- |
| anti-Desmin | Dako | human Desmin purified from skeletal muscle, known to recognise the avian protein | mouse monoclonal (IgG1) | HIER  pH 9 | 1:50-1:100 |
| MF20 | Developmental Studies Hybridoma Bank | light Meromyosin from chicken pectoralis muscle, detects all sarcomeric Myosins | mouse monoclonal (IgG2b) | - | 1:200 |
| anti-Myh7 | Sigma | partial human Myh7 peptide, 87% identity with corresponding chicken sequence | rabbit polyclonal | HIER  ph6 | 1:100 |
| anti-Myh15 | Sigma | partial human Myh15 peptide, 58% identity with corresponding chicken sequence | rabbit polyclonal | HIER  ph6 | 1:50 |
| anti-Troponin I (skeletal, slow) | Sigma | partial human Tnni1 peptide, 79% identity with corresponding chicken sequence | rabbit polyclonal | HIER pH6 | 1:50 |
|  |  |  |  |  |  |
| **secondary antibodies** | **supplier** | **antigenic determinant** | **type** | **antigen retrieval** | **dilution** |
| anti-mouse IgG+IgM (H+L)  horse radish peroxidase | Jackson Immuno | mouse IgG and IgM and light chains of other mouse Ig | goat | - | 1:200 |
| anti-mouse IgG (H+L)-  Alexa fluor 594 | Jackson Immuno | whole mouse IgG | goat | - | 1:200 |
| anti-rabbit IgG (H+L)-  Alexa Fluor 488 | Invitrogen/ Jackson | whole rabbit IgG | donkey | - | 1:200 |

Alsan BH, Schultheiss TM (2002) Regulation of avian cardiogenesis by Fgf8 signaling. Development 129, 1935–1943.

Christophorou NA, Bailey AP, Hanson S, et al. (2009) Activation of Six1 target genes is required for sensory placode formation. Dev Biol 336, 327–336.

Goulding MD, Lumsden A, Gruss P (1993) Signals from the notochord and floor plate regulate the region-specific expression of two Pax genes in the developing spinal cord. *Development* 117, 1001–1016.

Graham A, Lumsden A (1996) Interactions between rhombomeres modulate Krox-20 and follistatin expression in the chick embryo hindbrain. *Development* 122, 473–480.
